# Prevention of the Disrupted Enamel Phenotype in *Slc4a4-*Null Mice Using Explant Organ Culture Maintained in a Living Host Kidney Capsule

**DOI:** 10.1371/journal.pone.0097318

**Published:** 2014-05-14

**Authors:** Xin Wen, Ira Kurtz, Michael L. Paine

**Affiliations:** 1 Center for Craniofacial Molecular Biology, Herman Ostrow School of Dentistry, University of Southern California, Los Angeles, California, United States of America; 2 Division of Nephrology and Brain Research Institute, David Geffen School of Medicine at the University of California Los Angeles, Los Angeles, California, United States of America; Tufts University, United States of America

## Abstract

*Slc4a4-*null mice are a model of proximal renal tubular acidosis (pRTA). *Slc4a4* encodes the electrogenic sodium base transporter NBCe1 that is involved in transcellular base transport and pH regulation during amelogenesis. Patients with mutations in the SLC4A4 gene and *Slc4a4-*null mice present with dysplastic enamel, amongst other pathologies. Loss of NBCe1 function leads to local abnormalities in enamel matrix pH regulation. Loss of NBCe1 function also results in systemic acidemic blood pH. Whether local changes in enamel pH and/or a decrease in systemic pH are the cause of the abnormal enamel phenotype is currently unknown. In the present study we addressed this question by explanting fetal wild-type and *Slc4a4-*null mandibles into healthy host kidney capsules to study enamel formation in the absence of systemic acidemia. Mandibular E11.5 explants from NBCe1^−/−^ mice, maintained in host kidney capsules for 70 days, resulted in teeth with enamel and dentin with morphological and mineralization properties similar to cultured NBCe1^+/+^ mandibles grown under identical conditions. Ameloblasts express a number of proteins involved in dynamic changes in H^+^/base transport during amelogenesis. Despite the capacity of ameloblasts to dynamically modulate the local pH of the enamel matrix, at least in the NBCe1^−/−^ mice, the systemic pH also appears to contribute to the enamel phenotype. Extrapolating these data to humans, our findings suggest that in patients with NBCe1 mutations, correction of the systemic metabolic acidosis at a sufficiently early time point may lead to amelioration of enamel abnormalities.

## Introduction

The formation of hydroxyapatite (Hap) in tooth enamel during amelogenesis generates large amounts of protons [1]. It has been estimated that as many as 14 moles of hydrogen ions are released during the creation of 1 mole of Hap [1]. Buffering of the enamel matrix against extremely acidic pH conditions during amelogenesis is thought to be important in order to maintain an optimal extracellular matrix pH (slightly acidic to near neutral) for the enamel matrix degrading proteases to function efficiently, and prevent the acidic demineralization of Hap crystallites [2], [3]. The electrogenic bicarbonate cotransporter (NBCe1) is a major pH regulator and base transporter in many tissues, including kidney, pancreas, eye, brain, and intestine [4], [5], [6]. In humans, the gene encoding NBCe1 (*Slc4a4*) transcribes three variants (NBCe1-A, -B and -C) resulting from two distinct promoters and alternative splicing [7]. Two additional variants (NBCe1-D, and -E) have been reported in the mouse [8]. Mouse enamel-forming cells (ameloblasts) express the NBCe1-B variant [9]. Human patients with mutations in the *SLC4A4* gene have abnormal dentition and enamel defects [10], [11], [12]. Furthermore, the enamel of NBCe1^−/−^ mice is hypomineralized and mechanically weak, and exhibits an abnormal prismatic architecture [13].

We previously hypothesized that NBCe1 plays a major role in regulating enamel matrix pH, and is essential for the normal development of the dentition. In addition to the hypomineralized enamel phenotype, patients with mutations in *SLC4A4* have corneal opacities (band keratopathy), cataracts, glaucoma, severe proximal renal tubular acidosis, calcification of the basal ganglia, elevated serum amylase and lipase, and mental retardation [10], [11], [12]. *Slc4a4-*null mice (henceforth referred to as NBCe1^−/−^ mice) have a more severe phenotype with volume depletion and decreased survival [13], [14]. In NBCe1^−/−^ mice, blood pH was significantly reduced from pH 7.3 (wild type) to pH 6.9 [14]. Severe acidemia (blood pH <7.1–7.2) has been shown to predispose humans to cardiac arrhythmias, suppress myocardial contractility, impair oxygen delivery, induce venoconstriction, decrease total peripheral vascular resistance and blood pressure, and reduce hepatic blood flow [15], [16]. Changes in blood pH are also sometimes accompanied (depending on the efficiency of the pH regulatory processes and the specific cell type) by intracellular pH changes that on their own can have profound effects on the metabolic and biochemical pathways in cells [15], [16]. These extracellular and/or intracellular pH changes can potentially significantly affect a developing embryo.

Previous studies have shown that the enamel of NBCe1^−/−^ mice is hypomineralized with an abnormal prismatic architecture [13]. However, it is currently unknown whether this phenotype is due to the decrease in systemic pH due to proximal renal tubular acidosis during development, or whether instead the local loss of ameloblast NBCe1-mediated base transport directly impacts tooth development. In this study we circumvented the effect of the systemic metabolic acidosis by transplanting NBCe1*^−/−^* mouse embryonic day 11.5 (E11.5) mandibles underneath the kidney capsule of syngenic wild type (WT) adult male mice [17]. Using this approach, the transplanted mandibles are in direct contact with the renal cortex and kidney capsule, allowing for blood-derived nutrients to fully engulf the transplants. Since the transplant derives all nutrients from the host’s blood supply, any differences in the enamel phenotype between the NBCe1^−/−^, NBCe1^+/−^ or NBCe1^+/+^ mice could then be attributed to the localized effects of ameloblast-specific NBCe1-mediated functional activity. Our data indicate that mandibular explants, grafted into host kidney capsules for a period of 70 days, result in recognizable enamel morphology and mineralization. In fact, the enamel from the NBCe1^−/−^ explants was more highly mineralized and more structured than enamel from 12-day old NBCe1^−/−^ mice [13]. We conclude that during enamel development, extracellular pH has a significant influence on enamel mineralization and plays a key role in the enamel phenotype due to loss of NBCe1 function. Moreover, quantitatively, defective ameloblast NBCe1-mediated transport *per se* appears to be less of a factor in normal enamel matrix hydroxyappatite formation than normal extracellular blood pH.

## Materials and Methods

### Animal Study Approval

All methodologies and animal manipulations related to this study were approved by the University of Southern California’s (USC) Institutional Animal Care and Use Committee (IACUC).

### Timed Pregnancy

NBCe1 heterozygous mice (NBCe1^+/−^; Black Swiss genetic background [14] were mated and produced liters with three different genotypes (NBCe1^+/+^, NBCe1^+/−^ and NBCe1^−/−^). Two females were placed in a cage that contained one male and the females’ sperm plugs were checked early the following morning. Embryos were considered one-half day old at the time the plug was noted.

### Mandible Dissection from E11.5 Embryos

In mice, at E11.5 the tooth germs are present as the dental lamina and the enamel organ is yet to be established, thus no enamel matrix or mineralization is present [18]. Pregnant mice were euthanized when the embryos were 11.5 days old, and afterwards an incision into the abdomen of each female exposed their organs. Embryos were dissected out of the mice by holding the placenta with tweezers, and by making an incision into the embryonic sac. Using a plastic pipette with a wide opening, the extracted embryos were transferred to a clean dish that contained HANKS solution. Mandibles were dissected by cutting in between the first and second branchial arch, followed by cutting through the mouth opening. Next, the tissues surrounding the mandible were carefully trimmed away. Discarded embryonic tissue was used for genotyping using methodologies described previously [13], [14].

### Mandible Organ Culture Prior to Transplantation

To establish the organ culture, a triangular metal grid (one inch on each side) was first sterilized with 70% alcohol and then flamed. The grid was bent at the corners to fit properly and then securely placed over the inner well and conserve medium of the organ culture dish. Next, to maintain humidity, the organ culture dish was placed inside a cell culture dish supplied with water and gauze. Six-millimeter diameter supporting filter papers were prepared by punching Type AA 0.8 µm pore size filters (AABP-047-00, Millipore, Bedford) with a Miltex biopsy punch. The filters were rinsed five times with ddH_2_O, and then boiled in ddH_2_O for 5 minutes followed by a final rinse with ddH_2_O and 70% ethanol. The filters were dried in a laminar flow hood. Organ culture medium (BGJb medium [Invitrogen] supplemented with 0.1mg/ml ascorbic acid, 100U penicillin, 100 U streptomycin and 10% fetal bovine serum) was prepared just prior to culture. Filter papers were inserted slowly into the dish containing dissected organs to allow air to escape. Each organ was pushed over the filter paper and the filter was lifted out and placed on top of the grid. Just enough culture medium was added to clearly see a ring outside the filter. The organ culture was kept overnight in an incubator at 37°C with 5% CO_2_ while genotyping of the discarded embryonic tissues was performed [13], [14].

### Renal Grafting

The surgical procedures for kidney capsule implantations essentially follow the protocols of Drs. Brody, Young, and Cunha (http://mammary.nih.gov/tools/mousework/Cunha001/index.html) [17]. Briefly, 10-week-old male wild type mice were anesthetized by intraperitoneal injections with ketamine (100mg/kg) and xylazine (10mg/kg). The loss of palm pinch reflex was verified before starting each procedure. The mouse was shaved on the dorsal side, about 2 cm above its tail along the spine to mid back, and on each side of the spine about 1 cm in width. Seventy percent ethanol (and 30% ddH_2_0) was applied with sterilized gauze to remove loose hair. A vertical incision was made through the dermis along the spine, about 2 cm from the base of the tail to the top curve of the spine. The mouse was laid on its right side for operation on its left kidney, which can be identified by looking for a triangular, less dense area, with the spleen at its top left side and the spine at its right side. A 0.5 cm horizontal incision was made on the left side of the body wall, and the kidney was popped out of the incision by applying a little pressure to either side of the kidney. A small slit was made in the kidney capsule with sharp forceps, and a smooth close-ended Pasteur pipette was inserted under the capsule tangential to the kidney parenchyma to create a pocket. The mandible with the filter paper was transferred into the kidney capsule and the mandible was gently pushed deep into the pocket. The kidney was then guided back into the body cavity by pulling up on the two sides of the incision, and the host’s muscle layer was sutured closed (Ethilon 5-0 19mm 3/8 reverse cutting). Next, the incised skin was closed and clamped with stainless steel wound clips (reflex skin closure system, reflex 7), and the animal was kept warm on a heating pad to help recovery. Mandibles were left in the host for 70 days (10 weeks). To collect the cultured teeth, the host was euthanized and teeth were carefully dissected from the surrounding tissues.

### Control Mouse Strain Black Swiss 2-week Mandibular First Molar Included for Analysis Comparison

An *in situ* prepared 2-week old mandibular first molar was included in the study for comparisons with the explanted tooth germs. At 2-weeks the enamel of the mandibular first molars are fully mature, thus represent the likely upper limits of enamel density and mechanical properties.

### MicroCT (µCT) Analysis

The tooth samples (those grown in the kidney capsule and the *in situ* control teeth) were air-dried, scanned, and reconstructed on a SCANCO µCT 50 at the University of Southern California’s Molecular Imaging Center. The acquisition settings were: 90kVp, 44uA, 1200ms exposure (3 averages, 3600 ms exposure), 1000 projections per 180 degrees and 2 micron isotropic resolution (FOV ∼ 4.1×4.1×1−1.2 mm^3^, grid: 2048×2048×500–700). The acquisition proceeded for ∼ 3.6 hours depending on the sample size. The images were exported in dicom format from the SCANCO workstation. The volume/surface was rendered, enamel and dentin segmented and their mean intensity calculated in AVIZO 7.1.1.

### Statistical Analysis of Enamel and Dentin Density

Student’s t test was performed to compare measurements of enamel and dentin density between all groupings of NBCe1 mice (NBCe1^+/−^, NBCe1^+/−^ and NBCe1^−/−^). In addition, data was compared for the enamel and dentin densities between the *in situ* grown 2-week first molar and the NBCe1^+/+^ teeth grown in the kidney capsule. Raw data are presented in Table 1.

**Table 1:** Mean intensity of enamel and dentin for teeth cultured under the host kidney capsule, and compared to mature, *in situ* grown teeth.

		ENAMEL			DENTIN		
Genotype	Sample ID	Raw Data	Average	St. Dev.	Raw Data	Average	St. Dev.
*NBCe1+/+*	*1*	*6894*			*5388*		
*NBCe1+/+*	*7*	*10332*	*7916 (n = 3)*	*2101*	*5655*	*5437 (n = 3)*	*198*
*NBCe1+/+*	*2*	*6521*			*5268*		
**NBCe1+/−**	**4**	**7826**			**5500**		
**NBCe1+/−**	**6**	**7757**	**7826 (n = 3)**	**69**	**5420**	**5419 (n = 3)**	**81**
**NBCe1+/−**	**14**	**7894**			**5338**		
*NBCe1−/−*	*94*	*7361*			*5813*		
*NBCe1−/−*	*93*	*6866*			*5509*		
*NBCe1−/−*	*9*	*7834*	*7447 (n = 5)*	*366*	*5511*	*5558 (n = 5)*	*152*
*NBCe1−/−*	*10*	*7612*			*5408*		
*NBCe1−/−*	*15*	*5760*			*5553*		
**wild type ** ***(in situ)***	**A**	**9548**			**5889**		
**wild type ** ***(in situ)***	**B**	**10828**	**10,027 (n = 3)**	**698**	**5978**	**5900 (n = 3)**	**73**
**wild type ** ***(in situ)***	**C**	**9706**			**5834**		

**NOTES:**

Enamel *p* values: NBCe1^+/+^/NBCe1^+/−^  = 0.94; NBCe1^+/−/^NBCe1^−/−^  = 0.14; NBCe1^+/+^/NBCe1^−/−^  = 0.63; *in situ* wild type/NBCe1^+/+^  = 0.17.

Dentin *p* values: NBCe1^+/+^/NBCe1^+/−^  = 0.89; NBCe1^+/−/^NBCe1^−/−^  = 0.36; NBCe1^+/+^/NBCe1^−/−^  = 0.36; *in situ* wild type/NBCe1^+/+^  = 0.02.

### Scanning Electron Microscopy (SEM)

Samples were prepared and imaged by SEM as previously described [13].

## Results

**Figure 1:**
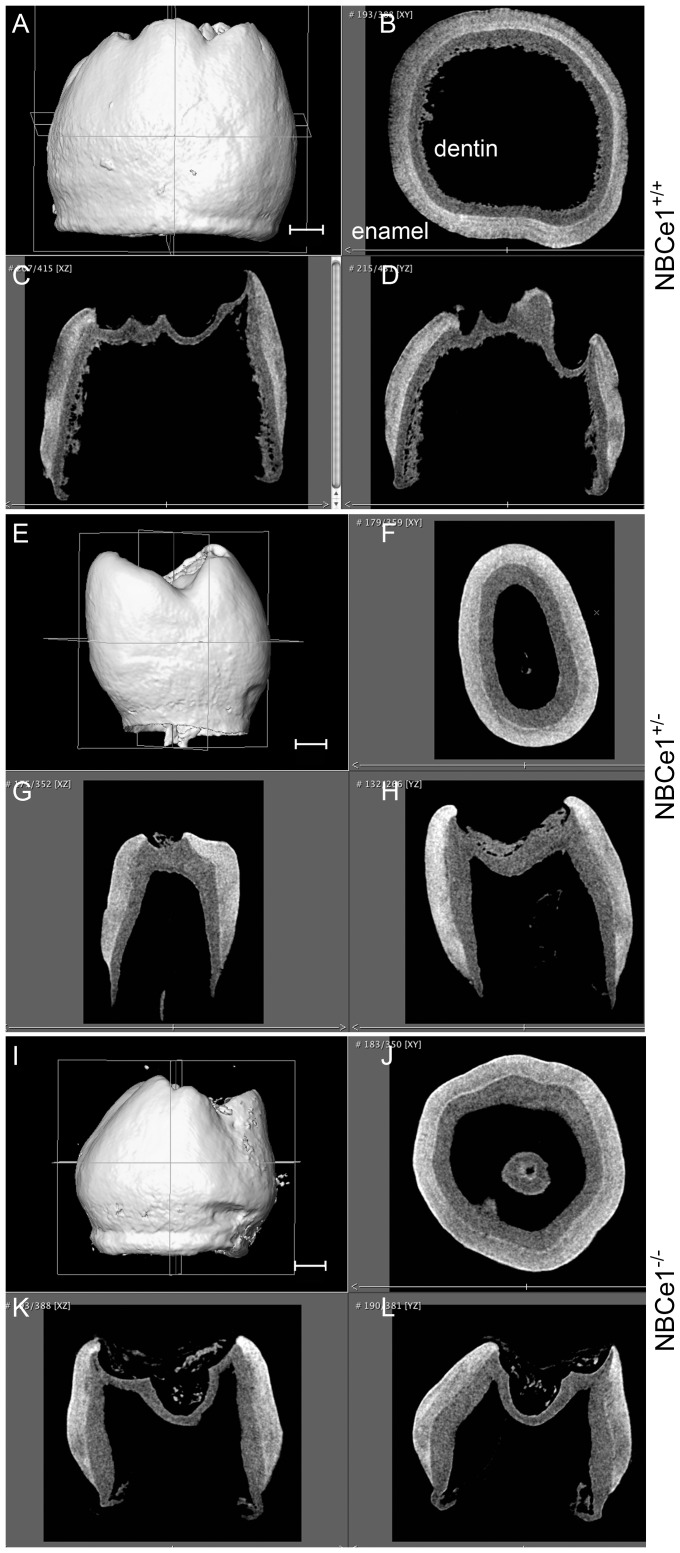
NBCe1^+/+^ (panels A–D), NBCe1+/− (panels E–H) and NBCe1^−/−^ panels (I–L) teeth at age E11.5+70 in host kidney capsules. Micro-CT three-dimensional (3D) images (panels A, E and I) and cross-sectional slices (panels B–D, F–H and J–L) of three typical examples of teeth grown underneath kidney capsule. Avizo software was used to analyze µCT data. Scale bars shown in panels A, E and I are 100.0 µm and are common to panels A–D, E–H and I–L respectively.

**Figure 2:**
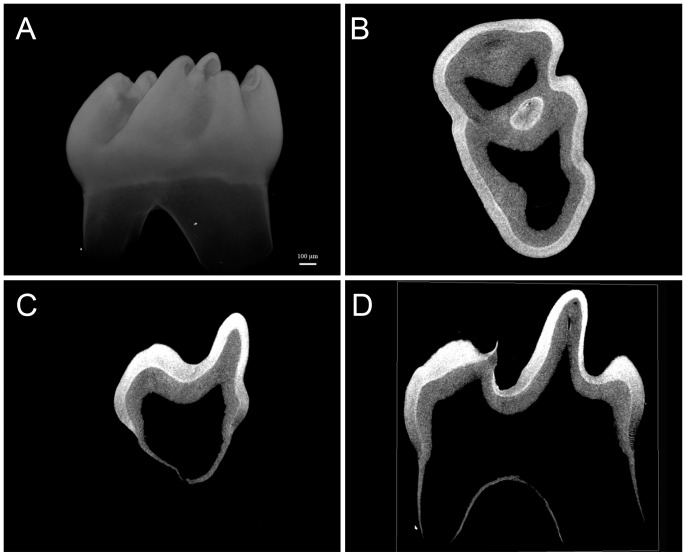
*In situ* derived 2-week old mandibular first molar. Micro-CT 3D image (panel A) and cross-sectional slices (panels B–D) of an *in situ* collected 2-week old mandibular first molar tooth used for comparison with the kidney capsule grown teeth. Avizo software was used to analyze µCT data. Scale bar shown in panel A is 100.0 µm and is common to panels A–D.

Fetal mouse mandibles were dissected at E11.5, transplanted underneath the kidney capsule and grown for 70 days (E11.5+70). The mandible was chosen for grafting to limit any damage to the tooth germ from direct contact with the surgical tools. All cultured teeth were isolated from the same pregnant female, and implanted into 10-week old sibling male hosts. After 70 days the teeth were carefully recovered from the explanted mandibles and scanned using microCT (µCT). Mandibles from E11.5 mice were chosen for explantation because they are of an ideal size and solidity for the operation, compared with mandibles from earlier embryonic stages. The recovered teeth, 2 - 4 teeth on average for each transplanted mandible, were disproportionately molars (first, second and third molars) with recognizable cusp morphology (Figure 1). One observation is that the overall morphology of the teeth is affected by the explant conditions with the overall mesiodistal and buccolingual dimensions being approximately half of that seen in in situ derived mandibular first molars (images seen in Figure 1 when compared to image in Figure 2). Incisor teeth were noticeably absent from the cultured mandibles. The reason for the smaller molar tooth dimensions, and the lack of incisors in explants is undetermined, but it may be due to their higher sensitivity to the space limitations and mechanical pressures present within the kidney capsule environment.

**Figure 3:**
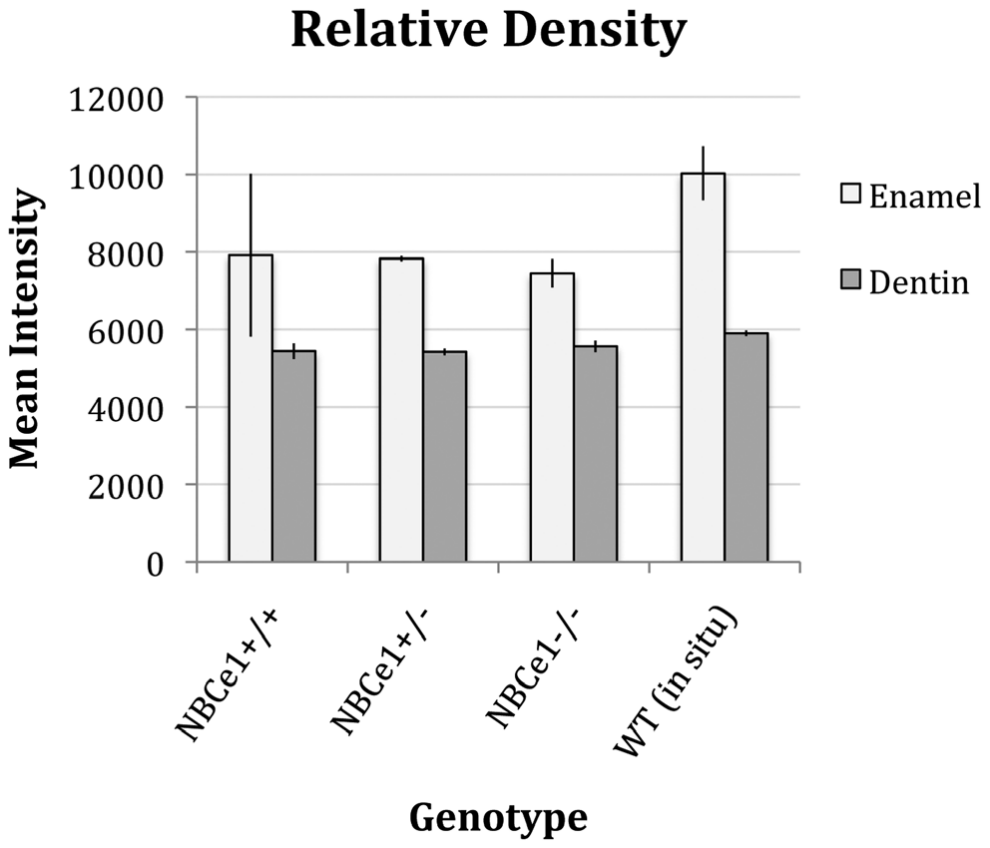
Enamel and dentin relative density measurements. The densities of teeth grown in the host kidney capsule, indicated by mean intensity value from µCT scanning, were compared across NBCe1^+/+^, NBCe1^+/−^ and NBCe1^+/−^ groups. Teeth imaged and used for analysis were NBCe1^+/+^ (n = 5), NBCe1^+/−^ (n = 3) and NBCe1^−/−^ (n = 5). The averages and standard deviations (error bars) were plotted. Two-tailed Student’s t tests were run and no statistically significant difference in either enamel or dentin density was identified between any of the groupings. Refer to Table 1 for raw data.

Visually, from the µCT images, the density of both the enamel and dentin of the cultured NBCe^−/−^ teeth at E11.5+70 was similar to that of the NBCe1^+/+^ and NBCe1^+/−^ teeth (typical examples of NBCe1^+/+^, NBCe1^+/−^ and NBCe1^−/−^ are shown in Figure 1). For NBCe1^+/+^, NBCe1^+/−^ and NBCe1^−/−^ cultured mandibles, the enamel was noticeably of greater density than the dentin (Figure 1, panels B–D, panels F–H and panels J–L), which was not the case for the enamel of 12-day old NBCe1 mutant mice examined previously [13]. It can be appreciated that the enamel of the NBCe1^+/+^ teeth cultured under the kidney capsule (Figure 1) appears less dense than the enamel of NBCe1^+/+^ teeth grown naturally (*in situ*) in the oral cavity (Figure 2) [13], however in the sample population analyzed, statistically this was not the case (Table 1, and Figure 3). While a larger sample size may aid with the interpretation, it is apparent that explanted teeth show enamel with a relatively wide variance (as indicated by the range of score and the standard deviation) of density while teeth grown *in situ* exhibit a relatively tight variance in enamel density (Table 1). Data obtained from 3 NBCe1^+/+^ teeth, 3 NBCe1^+/−^ and 5 NBCe1^−/−^ teeth showed no statistically significant differences in the density of either their enamel or dentin (Table 1 and Figure 3). This was in sharp contrast to what has been observed in NBCe1^−/−^ mice *in situ* [13], which displayed extremely poor mineralization of enamel when compared to either their NBCe1^+/+^ or NBCe1^+/−^ littermates [13]. There was a statistically significant difference in the dentin density between the NBCe1^+/+^ teeth grown in the kidney capsule when compared to the *in situ* grown teeth (*p*<0.05), despite the absolute density readings being similar (5437 vs. 5900 units respectively; Table 1). In summary, the enamel from cultured NBCe1^−/−^ mandibles has a greater density than that of non-cultured (*in situ* formed teeth) NBCe1^−/−^ teeth taken directly from the mutant mice [13].

**Figure 4:**
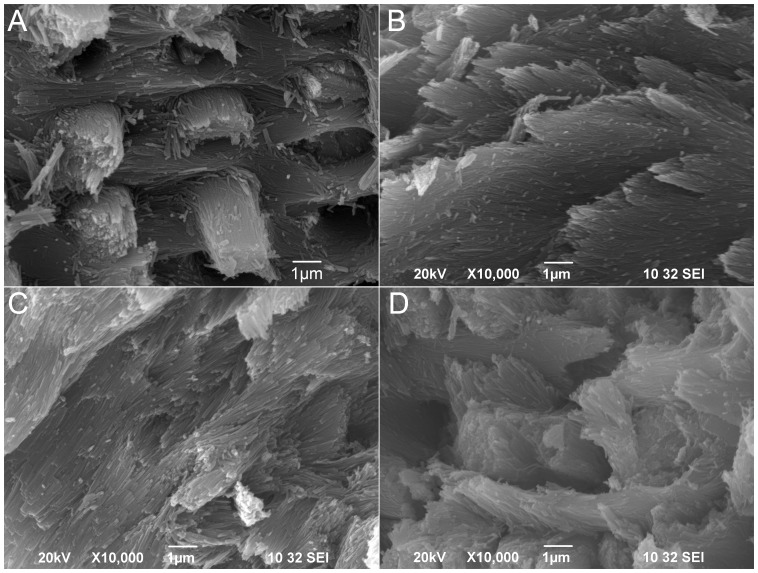
Scanning electron microscope (SEM) images of tooth enamel. Teeth were cracked open with a diamond knife and then coated with gold/platinum for SEM observation. Panel A is taken from a normal (wild-type) incisor taken from a mature NBCe1^+/+^ animal. Panels B, C and D are taken from the NBCe1^+/+^, NBCe1^+/−^ and NBCe1^−/−^ teeth, respectively, grown in the kidney capsule. Scale bars are included in each panel.

The architecture of the enamel was also examined using scanning electron microscopy (SEM). The enamel crystallites appeared to be of normal size in all NBCe1^+/+^, NBCe1^+/−^ and NBCe1^−/−^ animals, when compared to fully mature enamel formed *in vivo* (Figure 4, panels B, C and D when compared with panel A). However, the typical rod-interrod organization seen in naturally-grown teeth (Figure 4A) appeared somewhat disrupted in all teeth cultured under the kidney capsule. This disruption of the regular rod-interrod organization could be partially explained by the compromised enamel density found in cultured teeth, as observed by µCT. In addition, the kidney capsule environment may be lacking certain extracellular factors that are present locally in the jaw *in vivo* that contribute to enamel mineralization, thus a compromised structure could be expected.

## Discussion

During enamel development, ameloblasts are exposed to local changes in pH [19], [20] in addition to the more constant systemic pH. Changes in local enamel matrix pH are thought to play a key role in the normal development of enamel Hap [3]. The relative importance of the systemic pH on enamel development has not been previously studied. However, based on a single patient with persistent renal tubular acidosis of the proximal type (with a measured blood pH ∼ 7.1), it has been speculated blood pH can impact on enamel morphology [21]. In this study, we show for the first time that the abnormal phenotype in enamel from an animal model that manifests both abnormal local enamel matrix H^+^/base transport and an acidic systemic pH can be corrected in the presence of a normal systemic pH. Specifically, the enamel density of teeth from NBCe1^−/−^ mice was comparable to that of wild-type mice when grown in a wild-type kidney capsule milieu. Moreover, our results suggest that abnormal NBCe1 activity perturbing the local enamel matrix pH is not sufficient to disrupt enamel formation once the systemic pH is normalized.

The vascular supply to the enamel organ is established early during development. Early during development, prior to enamel or dentin formation, a vascular network is established within the stellate reticulum in close proximity to the inner enamel organ. As the tooth germ develops, the inner enamel epithelial cells differentiate to form a monolayer of ameloblasts that become highly polarized. At some stage during early tooth development, two to three layers of cells form the stratum intermedium between the ameloblast monolayer and stellate reticulum. The origins of the stratum intermedium cells are unclear, but there is evidence that part of the stratum intermedium cell population originates from the inner enamel epithelium [22]. It is thus likely that cells from both the inner enamel epithelium and the stellate reticulum contribute to the stratum intermedium cell population. Secretory ameloblasts are 5–6 µm in diameter and can be up to 100 µm in length; thus, the vasculature of the stellate reticulum can be separated from the forming enamel matrix by >100 µm (the total thickness of the ameloblast and stratum intermedium cell layers) [19], [23], [24]. While it has been judged that cells can survive a few hundred microns away from a capillary (a source of nutrients) [25], there is little information indicating what the relative contributions of the vasculature and the local cellular environment (ions secreted from the apical pole of ameloblasts) are in influencing mineralization once a mineralized tissue matrix has been established. There are, however, multiple *in vitro*, cell-free model systems where Hap “enamel-like” crystallites can form in an environment that is rich in enamel proteins [26], [27], [28], suggesting that once the enamel matrix is established, further ameloblast-specific activities may be negated provided the appropriate milieu is created. These and similar studies have led to opportunities to develop bio-inspired materials and to the science of biomimetics [29], [30].

Multiple disease states that impact the circulating levels of calcium, phosphate, fluoride, or bicarbonate are known to impact amelogenesis, and generally result in hypomineralized enamel with compromised mechanical properties. An example is hypophosphatasia (HPP), which can result in, amongst other pathologies, enamel hypoplasia [31]. In a mouse model, if HPP (including enamel hypoplasia) results from a deficiency of the tissue-nonspecific isoenzyme of alkaline phosphatase (ALPL) function, the enamel phenotype can be rescued with subcutaneously administered enzyme replacement therapy [32]. Multiple reports also link hypocalcemia and hypophosphatemia to bone, dentin, and enamel pathologies, including DiGeorge syndrome [33], vitamin D receptor (VDR)-associated rickets [34], [35], and phosphate-regulating endopeptidase homolog, X-linked (PHEX)-associated rickets [36]. There is also evidence in VDR mutant mice susceptible to rickets that many of the associated bone and dental (dentin and cementum) pathologies can be partially corrected via oral supplementation to correct the levels of circulating calcium and phosphate. However, in the VDR mutant mice there are conflicting reports as to whether the enamel pathologies are correctable [35], [37], [38], [39]. These data suggest that in certain genetic diseases that involve an abnormality in ameloblast ion transport and an alteration in blood chemistry, normalizing the abnormal blood chemistry may help minimize the enamel pathology if done at the appropriate time during tooth crown development. It is intriguing to recognize that the clearly-defined enamel pathology described in pRTA could be corrected if the surrounding environment is maintained at normal physiological pH levels. To extrapolate these mouse data to humans, the human permanent dentition starts to form 2–3 months after birth, and with the exception of the second and third molars, the crowns are fully mineralized by ∼ 7 years. It may be therefore feasible to treat pRTA individuals during their infant years with bicarbonate supplementation to lessen the impact of enamel disease that is directly associated with the acidic blood pH levels. Our data suggest that there is likely a finite window of time during enamel development when normalizing the local pH could be beneficial.

Our study also raises interesting questions regarding the relative contribution of the maternal circulation, the placenta, and the fetus to the acid-base status of the fetus at various stages of gestation [40]. Although this question has not been addressed in detail, it is known that kidney function gradually develops from conception to birth [40], [41], and the acid-base status of the fetus differs from the mother [40], [42]. Specifically, the bicarbonate concentration and pH of fetal blood is normally lower than maternal circulation in both early pregnancy and at term [42]. The cause of the fetal metabolic acidosis has been hypothesized to be due to enhanced fetal metabolic proton production [42]. Other factors may also be involved including the limited ability of the placenta to transfer protons to the maternal circulation and the limited capacity of the fetal kidney to generate “new” bicarbonate from organic anion metabolism. Regardless of the mechanism, it appears that placenta/maternal circulation are unable to compensate for the more severe metabolic acidosis that is likely present in NBCe1^−/−^ mice.

## Conclusion

In conclusion, mandibles taken from E11.5 survive in a host kidney capsule for at least 70 days, and the encapsulated teeth continue to develop with a recognizable morphology and Hap crystallites. In these explants, the enamel and dentin remain clearly distinguishable, although the enamel mineralization and morphology are compromised. An earlier study showed that NBCe1^−/−^ mice at postnatal day 12 have hypoplastic enamel [13]; however, under organ culture conditions (E11.5+70) much of the enamel pathology seen in NBCe1 mutant mice can be rescued. Our data demonstrate that blood pH plays a significant role in proper enamel formation, and represent the first instance of reversal of a phenotype due to loss of NBCe1 transport function. Whether the correction of the systemic pH can reverse the abnormal phenotype seen in other organs due to loss of NBCe1 remains to be determined.
